# Domain-general but not speech-specific auditory duration perception predicts pseudoword reading in adults

**DOI:** 10.3389/fnhum.2023.1241589

**Published:** 2023-09-14

**Authors:** Ana Rita Batista, Dinis Catronas, José Sousa, Vasiliki Folia, Nathercia Lima Torres, Susana Silva

**Affiliations:** ^1^Center for Psychology, Faculty of Psychology and Educational Sciences, Psychology Department, University of Porto, Porto, Portugal; ^2^Lab of Cognitive Neuroscience, School of Psychology, Aristotle University of Thessaloniki, Thessaloniki, Greece

**Keywords:** duration perception, reading skills, pseudoword decoding, speech-related duration perception, domain-general duration perception, mismatch negativity

## Abstract

Associations between reading performance and duration perception have been found both for domain-general and speech-specific duration perception. However, research seems limited to children and, critically, the predictive value of the two duration perception modalities has not been compared so far. In the present study we compared the weight of domain-general (comparison of time intervals defined by beeps) vs. speech-specific duration perception (pre-attentive EEG responses to consonants with different durations) as statistical predictors of reading in a sample of 46 neurotypical adults (18–43 years old) with 13 years of schooling on average. Reading included word and pseudoword decoding, as well as reading comprehension. We ran one regression model with domain-general and speech-specific duration perception as predictors for each of the three reading skills. Pseudoword decoding was the only reading skill that was significantly predicted by duration perception, and this happened for domain-general duration perception only. A complementary analysis adding 26 typically developing and 24 dyslexic adults to the main sample (n = 96 in total) showed the same pattern of results in dyslexics, but not in added controls. Our findings strengthen the idea that duration perception is important to phonological encoding and its use in grapheme-to-phoneme conversion, given that only pseudoword decoding was predicted by the interval comparison task. The irrelevance of speech-specific duration perception tones down the possibility that accurately perceiving the length of speech sounds is crucial to skilled reading.

## 1. Introduction

Time perception abilities have been found to correlate with reading skills (e.g., Corriveau et al., 2007; Plourde et al., 2017; Casini et al., 2018). Time perception is the ability to estimate, discriminate and compare the time position (“when?”) and length (“how long?”) of events. It can be based either on a regular unit used as reference (beat-based time perception) or rely on the absolute, beat-unrelated processing of time intervals (duration perception, see Teki et al., 2011). Time perception should not be confused with temporal processing – i.e., temporal order and sequence processing (Thoenes and Oberfeld, 2017) – which has been pointed out as a core deficit in dyslexia by the temporal processing hypothesis (De Martino et al., 2001). Typical temporal processing tasks involve identifying or recognizing sequential events, e.g., quickly changing sounds or images (like flickering stimuli), while in time perception tasks the target is time itself. In the present study, we were concerned with the relation between time (specifically duration) perception and reading.

The possibility that accurate duration perception is necessary for reading follows at least two alternative hypothetical paths, both referring to phonological processing as a potential link between time perception and reading. One is based on the importance of ordinal prediction – i.e., anticipating what comes next in speech and/or text. This general approach has been put forward by the Temporal Sampling Framework (TSF). According to the TSF, the brain-oscillatory correlates of beat perception (i.e., periodic activity in the brain) would be responsible for effective speech encoding at multiple levels (phonemes, syllables, stress accents), and this would improve ordinal prediction for speech (Hickey et al., 2020). As oscillatory activity in the brain synchronizes with (entrains to) incoming speech units at the relevant frequency bands (e.g., gamma oscillations, above 30 Hz, synchronizing with incoming phoneme sequences), the brain ‘knows when’ each new unit will arrive, increasing temporal prediction. Increased temporal prediction increases attention to speech units, encoding is enhanced (Goswami, 2018), and so is ordinal prediction (knowing “what comes next”). In this view, time perception for all sorts of periodic (music-like) or quasi-periodic (speech-like) events – domain-general duration perception – would be important.

A second hypothetical link between duration perception and reading is that duration perception allows encoding speech units whose identity depends on length (Plourde et al., 2017). For instance, stop consonants differ from one another in time-related properties like voice onset time (e.g., Kent and Read, 2002), and listeners must acquire these contrasts throughout language acquisition (Saloranta et al., 2020). From this viewpoint, time perception for speech sounds – speech-specific duration perception – would be critical.

Research on the relation between duration perception and reading skills has focused both on domain-general and speech-specific duration perception. Oddball paradigms (reaction to deviant sounds in a stream of standard, constant stimuli) have been widely used to test discrimination between shorter and longer sounds. The Mismatch Negativity (MMN) paradigm is one such approach, where pre-attentive (passive) processing of deviants shows up in the EEG as an increased early negativity with anterior-central topography (Näätänen and Alho, 1997). Again, it is important to distinguish between MMN studies measuring time discrimination (short-long discrimination, our focus) and those measuring temporal discrimination (identifying sounds that unfold in rapid succession, as it typically happens in MMN paradigms). The latter have also been used in dyslexia, but with a different purpose – test for the temporal processing deficit hypothesis on reading disabilities (Gu and Bi, 2020).

Regarding domain-general duration perception, MMN studies using shorter vs. longer tones as standard vs. deviant stimuli have shown differences between dyslexic and control children (Corbera et al., 2006; Huttunen et al., 2007). Behavioral studies with children found significant associations between the perception of tone length and various reading or reading-related domains such as phonological awareness (Corriveau et al., 2007; Degé et al., 2015; Casini et al., 2018), reading speed and/or accuracy of words (Corriveau et al., 2007; Plourde et al., 2017; Casini et al., 2018), pseudowords (Plourde et al., 2017) and non-words (Corriveau et al., 2007; Casini et al., 2018), or reading comprehension (Corriveau et al., 2007; Plourde et al., 2017; Casini et al., 2018). In sum, considerable evidence for a link between domain-general duration perception and reading skills is available, at least in children.

Concerning speech-specific duration perception and reading, Pennala et al. (2010) investigated the association of vowel length (70 vs. 110 vs. 130 ms) behavioral discrimination with reading accuracy, reading speed, and spelling accuracy in reading-disabled vs. control children. The results showed that discrimination in reading-disabled children was lower than in the control group and explained the unique variance of spelling accuracy after considering the variance in verbal short-term memory, phonological memory, and naming speed. No relation was seen in the control group. Most studies in this area used oddball paradigms – either pre-attentive (MMN-elicitors) or attentive (P3-elicitors) – coupled with EEG recordings. Lovio et al. (2010) presented different vowel durations (vowels /e/ or /i/ with different lengths preceded by plosives) to dyslexic vs. control children in a passive (pre-attentive) oddball task. The duration of the syllable was 170 ms and deviant syllables differed by −70 ms or + 100 ms based on vowel length manipulations. Children with dyslexia showed lower MMN amplitudes to length deviants.

Like vowel length, Voice Onset Time (VOT) is a phonetic feature that relates to duration perception. VOT is the interval between the noise burst produced *via* the initial articulatory release of a stop consonant and the onset of the first glottal pulsing of the subsequent vowel (Lisker and Abramson, 1964; Kent and Read, 2002). VOT allows distinguishing between voiced (/b/, /d/, /g/) and voiceless (/p/, /t/, /k/) stops in English and in a number of other languages (Abramson and Lisker, 1973; Hutters, 1985; Ito and Mester, 1986). The voiced stop consonants have shorter VOT values (−20 ms to +20 ms) than voiceless ones (25 ms to 100 ms) (Lisker and Abramson, 1964). VOT has been widely used as an acoustic correlate of voicing contrasts among the world’s languages.

Chobert et al. (2012) ran a mismatch negativity (MMN) protocol to test for the pre-attentive perception of syllables varying in pitch, vowel duration or VOT. They employed a sequence of syllables including standards (/ba/) and deviants (/pa/). Deviants differed from standards in vowel frequency, duration, and Voice Onset Time (VOT). Dyslexic children showed lower MMN amplitudes in response to VOT time and vowel duration, but not in response to pitch variation. In contrast to the above-mentioned findings, older studies did not highlight the relation between duration perception and reading. Kraus et al. (1996) found that difficulties in perceiving speech contrasts other than length may contribute more to reading difficulties in children. The authors used a passive oddball paradigm to examine behavioral and EEG discrimination (MMN amplitudes) of both spectral changes (/da/ vs. /ga/) and temporal changes between two syllables (/ba/ vs. /wa/, contrasting in the duration of formant transitions) in children with learning problems (poorer performance on reading measures vs. controls). While the discrimination of spectral contrasts was clearly different across groups, the same did not apply to duration contrasts. In a similar vein, Baldeweg et al. (1999) tested the association of pitch and duration discrimination with reading performance, employing both word and non-word reading. Behavioral discrimination and EEG responses (ERP latencies) for pitch contrasts correlated with reading errors for both words and non-words, but discrimination of duration contrasts did not.

In sum, while studies with children provide ample evidence for the importance of domain-general duration perception to reading, the role of speech-specific duration perception is yet unclear. One way to address this problem is to compare domain-general vs. speech-specific duration perception regarding the relation with reading. To our knowledge, this has not been done yet. On the other hand, the scarcity (or possible absence) of studies with adults minimizes the opportunities to clarify whether the association is based on phonological processing. While children may be expected to rely substantially on phonological encoding to read, neurotypical adults are supposed to do so only for non-words or pseudowords (Baldeweg et al., 1999).

In the present study, we investigated the relation between reading and time perception – including both domain-general and speech-specific duration perception – to determine which modality weights more on the reading skills of adults. To that end, we asked neurotypical adult readers to perform a behavioral interval-comparison task with beep sequences (domain-general duration perception), a pre-attentive VOT discrimination task with EEG recordings (speech-specific), and reading tasks relating to word decoding, pseudoword decoding and reading comprehension. Since our results pointed to an association between domain-general duration perception and pseudoword reading, we did a complementary analysis where we added data from another study of ours (Catronas et al., 2023) to enlarge our sample, increase statistical power, and get more reliable results.

We predicted that the relation between duration perception and reading in neurotypical adults would not go much beyond pseudoword decoding skills because, unlike children, phonological processing (grapheme-phoneme conversion) in adults is more likely to be limited to pseudoword reading (Baldeweg et al., 1999). For the same reason, we did not expect significant links between duration perception and reading comprehension, even though these have been found in children (Corriveau et al., 2007; Plourde et al., 2017; Casini et al., 2018). As for the weight of domain-general vs. speech-specific duration perception in reading, available findings for children suggest that the former may be more important. Since we had no reason to predict that adults would be different in this matter, our hypothesis was that domain-general, more than speech-specific duration perception, would be associated with reading, mainly with pseudoword decoding. The increased importance of domain-general duration perception would be consistent with the possibility that efficient entrainment to speech sounds (TSF-based mechanism) is more important to reading than accurate perception of phoneme length.

## 2. Materials and methods

### 2.1. Participants

*A priori* power analyzes for the EEG MMN study (two within-subjects conditions – standard and deviant) pointed to a minimum of 34 participants to allow capturing a medium effect size with 80% power and 0.05 of alpha error probability. An equivalent approach to power in a regression analysis with two predictors of reading (domain-general vs. speech-specific), indicated a minimum of 43 participants.

Fifty-four European Portuguese native speakers agreed to take part in this study. All participants gave their informed consent according to the declaration of Helsinki. The project was approved by the ethics committee from the Faculty of Psychology and Educational Sciences at University of Porto (Ref. 2022/01-10).

Data from eight participants were excluded from the analysis, either because their EEG contained more than 30% of contaminated trials (*n* = 3), or due to technical problems during data collection (*n* = 5). The final sample included 46 participants (35 women), with a mean age of 21.2 years (*SD* = 4.64; range 18–43) and 13.6 years of schooling (*SD* = 2.23 years; range 11–20 years). None of the participants reported hearing, cognitive or neurological problems. Reading skills were not part of inclusion or exclusion criteria, but we made a post-test check of participants’ reading profiles against norms.

Since our results were in line with the hypothesis that domain-general duration perception predicts the accuracy of pseudoword reading, we tested this association further with an enlarged sample to increase power and see whether evidence could be strengthened. This complementary analysis was conducted by adding data from participants in a previous study of ours (Catronas et al., 2023). The goal of that study was to understand if impairments in visual time perception of dyslexics were secondary to visual problems or not. The target of this study was visual time perception, but the same auditory time perception task with beeps we used here was also administered by then. Twenty-four dyslexics (22 female, 4 male, age: M = 24.8, *SD* = 7.8; schooling: M = 14.7, *SD* = 2.43) and 26 controls (21 female, 4 male, age: M = 23.5, *SD* = 7.47; schooling: M = 14.9, *SD* = 2.43) took part in this study In total, we had 96 participants. According to sensitivity power analyzes, the previous 46-participants sample was able to capture an effect size (f2) of 0.13 (already in the small-to-medium range) within a two-predictor regression, while the current sample was sensitive to effects as low as 0.11 (three predictors: domain-general duration perception, study and group).

### 2.2. Instruments

To measure word and pseudoword decoding skills, we used the 3DM test (adult version) as included in the ADLER battery (Adult DysLExia Reading battery, Faísca et al., 2019). Participants’ task consisted of reading aloud four lists of words (high-frequency, low-frequency, consistent, inconsistent) and one list of pseudowords. For each list, participants were asked to read the maximum number of items as accurately as possible in 30 s. Reading speed per list was computed as the number of accurate items divided by 30 (items per second). Scores for words (4 lists) were aggregated into a single one, representing word decoding, to be compared with pseudowords.

For reading comprehension, we used the 1 min – TIL (Reading Age Test). TIL is a short test validated by Fernandes et al. (2017), based on a version for children created by Santos and Castro (2008). The instrument comprises 36 sentences, arranged in columns, each sentence missing the last word. Participants were asked to choose the word that adequately completed each sentence from five alternatives, and to respond as fast and accurately as possible within a 1-min limit. The score was obtained by summing all correct responses (range 0–36).

### 2.3. Stimulus materials

To quantify domain-general duration perception, we asked participants to classify sequences of three 50 ms beeps separated by two intervals as either speeding up or slowing down, depending on whether the first or the second interval was the longest. We created 16 sequences, half speeding up and half slowing down, matching the two halves for interval content (intervals were the same but in reverse order, Table 1). The 16 sequences consisted of 16-bit mono audio files at 44.1 kHz sampling frequency. They had a length ranging between 467 and 933 ms and were preceded by a 200 ms pre-stimulus silence. For speed up sequences, the first interval ranged from 133 ms to 733 ms and the second from 167 to 433 ms. The order of intervals was reversed for slow down sequences. Table 1 shows the values of the first and second interval times for each sequence, as well as the difference between them.

**Table 1:** Time structure of beep sequences used in the duration perception task (ms).

Type	Interval 1	Interval 2	Difference	Type	Interval 1	Interval 2	Difference
Slow down	300	433	−133	Speed up	433	300	133
Slow down	167	300	−133	Speed up	300	167	133
Slow down	433	467	−34	Speed up	467	433	34
Slow down	167	733	−566	Speed up	733	167	566
Slow down	300	467	−167	Speed up	467	300	167
Slow down	134	434	−301	Speed up	433	134	299
Slow down	233	534	−301	Speed up	534	233	301
Slow down	433	500	−67	Speed up	500	433	67

**Figure 1:**
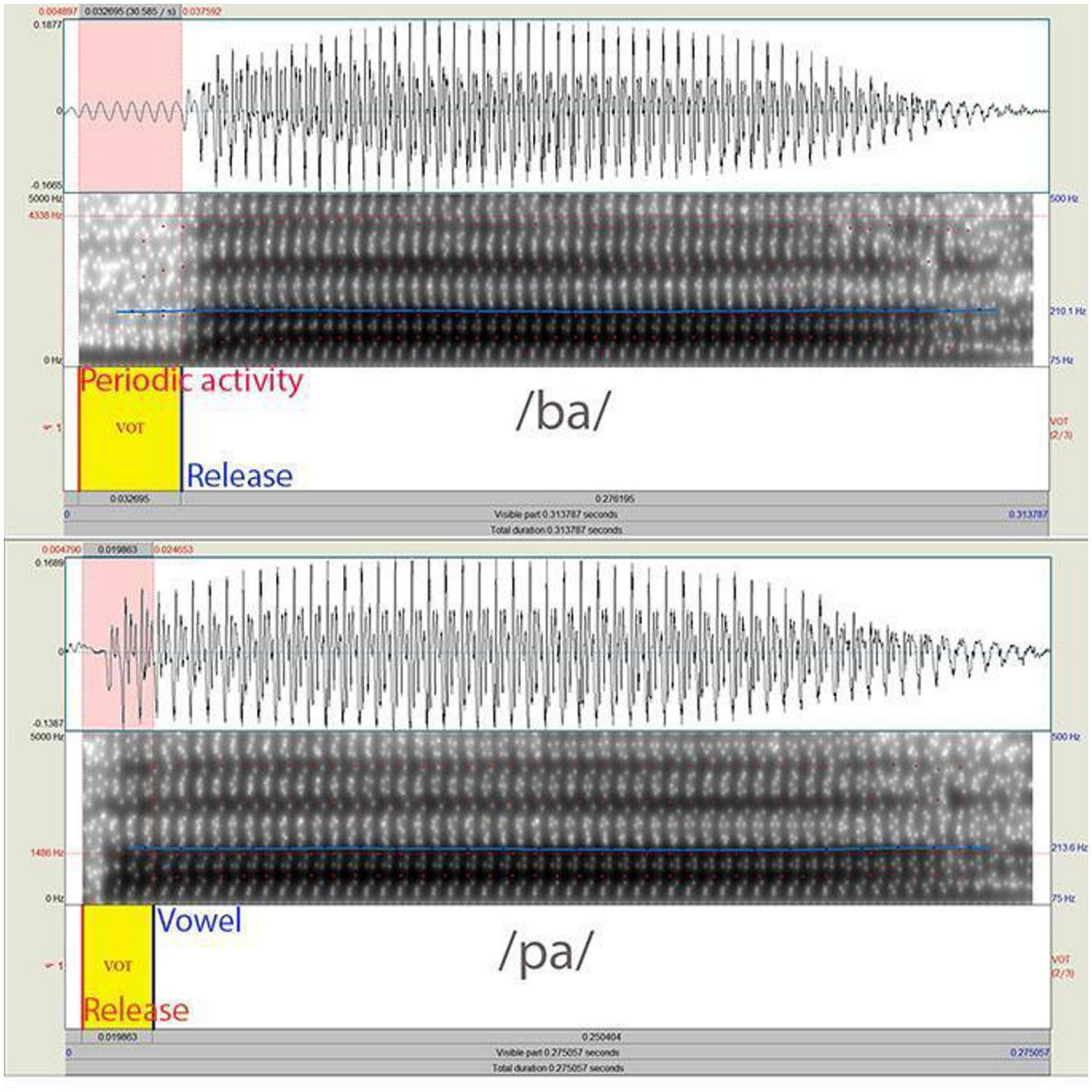
Acoustic structure of the syllables /ba/ and /pa/ used in the oddball task.

For the mismatch negativity (MMN) oddball task addressing domain-general duration perception, we used the syllables /ba/ and /pa/. One half of the experiment used /pa/ as standard, and the other /ba/ as standard. The syllables were produced by a female European Portuguese native speaker in a sound booth, and digitally recorded at 24 bit and a sampling rate of 48 kHz. After recording, we normalized the files to +70 dB rms and made minor editions of the pitch curves with Praat software^1^ to make sure that pitch was equivalent across the two syllables. Acoustic analyzes showed no relevant differences between /ba/ and /pa/ in formant- or pitch-related properties. Concerning formants, F1 average values were 808/824 Hz respectively, 1,468/1462 Hz for F2, 2,957/2946 Hz for F3 and 4082/3966 Hz for F4. The mean pitch was 211 Hz for both (Figure 1). As expected, VOT values were negative (−31 ms) for /ba/ (onset of release/burst as shown in waveform and spectrum minus onset of periodic activity from vocal folds) and positive (+ 19) ms for /pa/ (onset of the vowel’s second formant minus onset of release/burst, see Lousada et al., 2010, p. 33). Also, in line with the literature, the post-release period was shorter for the syllable with the voiceless /p/ consonant, making the length of the two syllables slightly different (313 ms for /ba/ vs. 275 for /pa/). Even though the syllables differed in more than one time-related property (VOT, vowel length and global length), the length-unrelated dimensions were kept constant, and we could be confident that duration was correctly manipulated.

### 2.4. Procedure

The experimental session included two different blocks: EEG (MMN task) and behavioral (three tasks: word/pseudoword reading, reading comprehension and duration perception from beep sequences). Both the order of blocks and the order of behavioral tasks were counterbalanced across participants. Data collection took place in an acoustically shielded room and lasted around 1 hour (EEG preparation included).

Before the beginning of the EEG recordings, participants were instructed to watch silent cartoon movies. To ensure they would be focused on the videos and not on the acoustic stimuli, they were told that we would ask them three questions about the movies later on. Half the participants started with /pa/ as standard and then followed into /ba/ as standard. The other half did the opposite. In total, participants listened to 2,400 stimuli, each part including 1,056 standard stimuli and 144 deviant stimuli (three was the minimum number of the standard stimuli between each deviant and 16 was the maximum). Syllables were separated by a 600 ms pause between stimuli. The onset time of the audio file was adjusted across standards and deviants such that the release of both occurred at 600 ms time intervals. Participants felt a regular beat subtending stimulus onset and were thus prevented from using irregular onsets as shortcuts for detection. Therefore, the /ba/ files had to be anticipated 50 ms because the release occurred later in the file.

In the interval comparison task, participants were asked to judge if each of the 16 sequences was speeding up (second interval shorter than first) or slowing down (second interval longer than first), pressing two different keys on the computer keyboard. After the instructions, participants did two practice trials and were clarified about possible doubts. Sequence presentation was randomized every time the experiment ran. Counterbalancing for response keys across participants was achieved by switching the label of the key numbers on the computer keyboard for half of them.

### 2.5. EEG recording and preprocessing

An electrode cap with 64 active channels was placed on participants’ scalp (10–20 system, FP1, FPz, FP2, AF7, AF3, AFz, AF4, AF8, F7, F5, F3, F1, Fz, F2, F4, F6, F8, FT7, FC5, FC3, FC1, FCz, FC2, FC4, FC6, FT8, T7, C5, C3, C1, Cz, C2, C4, C6, T8, TP7, CP5, CP3, CP1, CPz, CP2, CP4, CP6, TP8, P9, P7, P5, P3, P1, Pz, P2, P4, P6, P8, P10, PO7, PO3, POz, PO4, PO8, O1, Oz, O2, Iz). Three additional electrodes were placed: two at the mastoids for later re-referencing, and one under the left eye to record vertical eye movements (VEOG). The EEG data was collected at 512 Hz sampling rate. The quality of the signal was checked and kept under the system-recommended thresholds. Participants sat in a comfortable chair while watching cartoon movies from a tablet.

To preprocess EEG data, we used the Fieldtrip toolbox (Oostenveld et al., 2011) for MATLAB. One-minute epochs locked to stimulus onset were extracted for standards vs. deviants. Based on visual inspection of the vertical (face electrode referenced to frontal electrode) and horizontal EOG (frontolateral electrodes referenced one to the other), we marked the trials with vertical and horizontal eye movement artifacts. Trials containing other types of artifacts and defective channels were also detected through variance inspection. All these trials were rejected, and the channels were removed by interpolation using the nearest neighbor averaging. The remaining trials were baseline-corrected (200 ms pre-trigger), detrended, re-referenced to the mastoid electrodes, and band-pass filtered between 0.01 and 30 Hz. We averaged these preprocessed 1,200 ms (1,000 ms + baseline) trials per condition and subject, and then we did a grand average.

### 2.6. Statistical analysis

Within behavioral data analysis, participants’ reading scores (dependent variables) were inspected for possible deviations from test norms to make sure we were dealing with a neurotypical sample. For the beeps task (domain-general duration perception), we computed d’ scores (discrimination index), Stanislaw and Todorov (1999) per subject to be later inserted as predictors of reading measures. D′ scores were first analyzed for significant differences from (higher than) zero.

**Figure 2:**
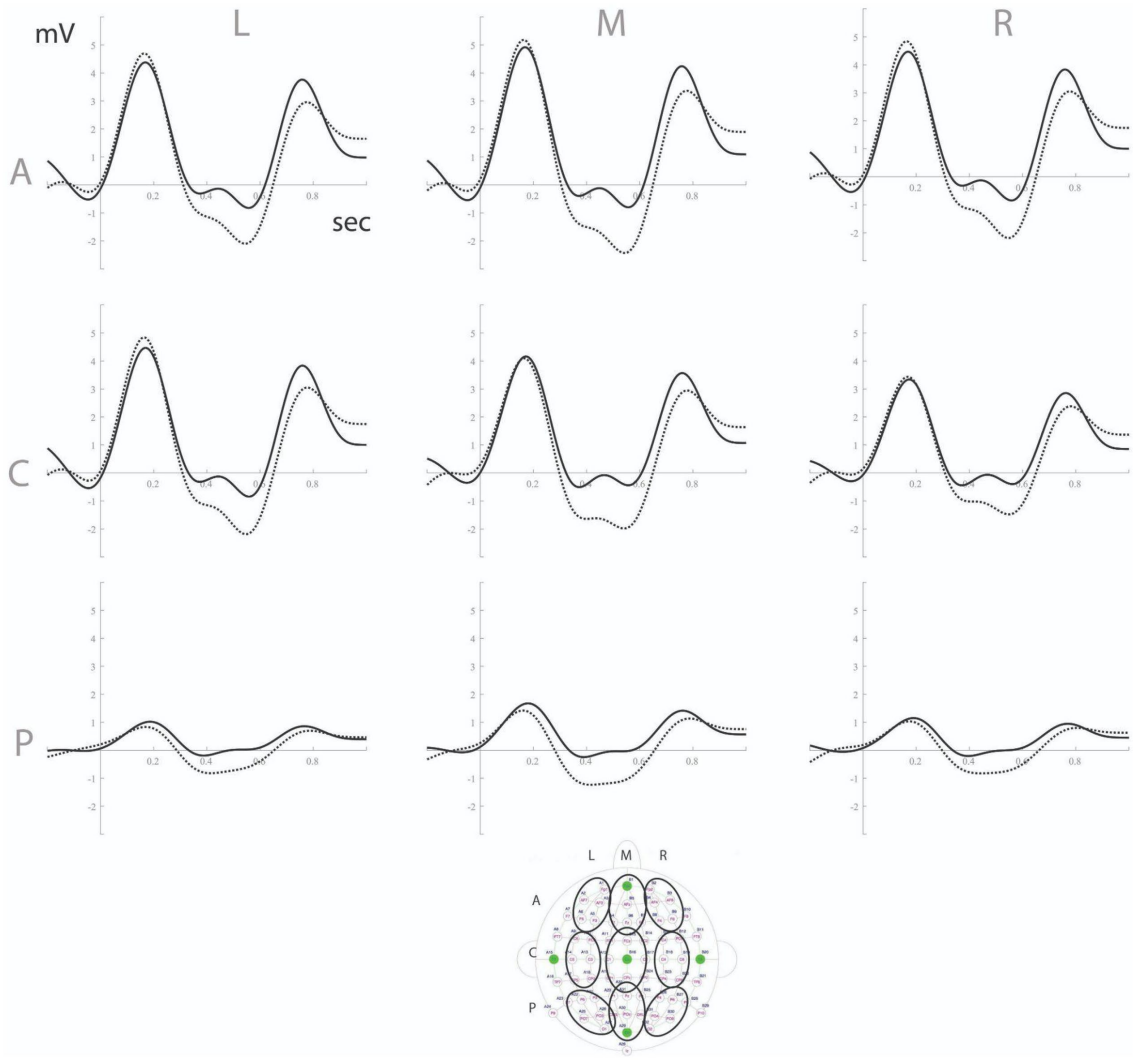
ERPs to standard (solid line) vs. deviant (dashed line) syllables in the 9 regions of interest (see below for precise identification of electrodes in each region). L, left; M, mid; R, right; A, anterior; C, central; P, posterior.

To analyze EEG data, we extracted three-time windows for statistical analysis based on Cluster Randomization Analysis (Maris and Oostenveld, 2007): a target window between 350 and 700 ms showing a significant negative cluster consistent with an MMN component, as well as the adjacent time windows (0–350 and 700–1,000 ms). Time-averaged voltage values were extracted per subject and region of interest (total of 9 regions, considering caudality and laterality: anterior, central, posterior x left, mid, right, see Figure 2) for the chosen time window. Repeated measures ANOVAs with condition (standard vs. deviant), caudality (three levels) and laterality (three levels) as factors were run for all three windows. In cases of sphericity violations, Greenhouse Geiser corrections were made.

We then investigated whether speech-specific (magnitude of MMN effect) and/or domain-general duration perception (interval comparison with beeps) predicted reading measures (3DM word decoding, 3DM pseudoword decoding and TIL), by means of regression models based on the enter method. To that end, the MMN effect (standard – deviant) as well as domain-general duration perception (beeps) were inserted as predictors of reading measures. For the complementary analysis (*n* = 96) on the relation between domain-general duration perception (predictor) and reading, we added study (current study vs. previous) and group (dyslexics, from previous study, vs. controls, from both studies) as predictors. We considered the main effects of these variables as well as their interactions with the predictor (beeps task). The presence of significant interactions was followed by correlational analyzes to investigate the beeps-reading relationship across groups, studies and reading measures.

The critical alpha value was set to 0.05 in all analyzes. When deviations from normality were present, we used non-parametric alternatives. For regression models, assumptions regarding autocorrelations, variance inflation factors and tolerance were checked.

## 3. Results

### 3.1. Reading measures and domain-general duration perception (behavioral)

Reading measures showed no relevant deviations from normative values (Table 2), indicating that participants had typical reading skills.

**Table 2:** Reading measures compared to normative values.

	Participants	Norms	Difference	Participants vs. M_norm_ + SD	
	*M ±* SD	*M ±* SD		*t*	*p*
Word decoding (correct/s)	1.87 ± 0.21	1.76 ± 0.24	0.11	−4.334	1.000
Pseudoword decoding (correct/s)	1.30 ± 0.24	1.23 ± 0.22	0.07	−473.141	1.000
Reading comprehension (TIL, max. 36)	17.93 ± 3.11	15.60 ± 2.70	2.30	−0.797	0.785

M, mean; SD, Standard Deviation; TIL, Teste de Idade de Leitura (reading age test).

Due to deviations from normality, we used the Wilcoxon signed-rank test to examine whether d´ values for domain-general duration perception (*M* = 1.67, *SD* = 1.94, no ceiling effects) were significantly higher than zero. The results showed significant discrimination between slow down and speed up sequences, *Z*(45) = 945. *p* < 0.001, highlighting participants’ ability to compare time intervals accurately. For a full report of descriptive values, please see Supplementary Table SA1.

### 3.2. Speech-specific duration perception (EEG)

Between 350 and 700 ms, the repeated measures ANOVA showed a significant interaction between condition and caudality on voltage values, *F*(1.13,51.3) = 5.75, *p* = 0.017, *η_p_^2^* = 0.11. We also observed a condition x laterality interaction, *F*(2,90) = 30.6, *p* < 0.001, *η_p_^2^* = 0.41. Post-hoc analysis showed that the MMN effect (deviant < standard) was widespread in the scalp, despite local variations in magnitude: anterior region, *F*(1,45) = 20.5, *p* < 0.001, *η_p_^2^* = 0.31; central, *F*(1,45) = 39.7, *p* < 0.001, *η_p_^2^* = 0.47; posterior, *F*(1,45) = 45.6, *p* < 0.001, *η_p_^2^* = 0.50; left region, *F*(1,45) = 27.1, *p* < 0.001, *η_p_^2^* = 0.38; mid, *F*(1,45) = 46.2, *p* < 0.001, *η_p_^2^* = 0.51; right, *F*(1,45) = 25.0, *p* < 0.001, *η_p_^2^* = 0.36 (see Figure 2).

For the 0–350 ms time window, the repeated measures ANOVA showed a significant interaction between condition and laterality, *F*(1.58,71.1) = 9.46, *p* < 0.001, *η_p_^2^* = 0.17. Post-hoc analyzes of condition effects per laterality level showed non-significant results for all levels (*p*s > 0.17). The 700–1,000 ms time window revealed a significant condition x caudality interaction, *F*(1.17,52.6) = 7.48, *p* = 0.006, *η_p_^2^* = 0.14, and a condition x laterality interaction, *F*(1.66,74.8) = 3.36, *p* = 0.049, *η_p_^2^* = 0.069. Post-hoc analysis showed a condition effect (deviant > standard) located in the anterior, *F*(1,45) = 9.35, *p* = 0.004, *η_p_^2^* = 0.17, and central, *F*(1,45) = 8.47, *p* = 0.006, *η_p_^2^* = 0.16, areas of the scalp, and at left, *F*(1,45) = 4.63, *p* = 0.037, *η_p_^2^* = 0.09, mid, *F*(1,45) = 8.73, *p* = 0.005, *η_p_^2^* = 0.16, and right, *F*(1,45) = 10.30, *p* = 0.002, *η_p_^2^* = 0.19, regions.

In sum, the 350–700 ms time window showed a component that was consistent with the MMN, despite the longer-than-typical latency (mean peak latency was 557 ms, *SD* = 143 ms). The preceding window exhibited no relevant activity, and the last window showed increased positivity for deviants (inconsistent with MMN, but still an index of length discrimination).

### 3.3. Domain-general vs. speech-specific duration perception as predictors of reading

**Figure 3:**
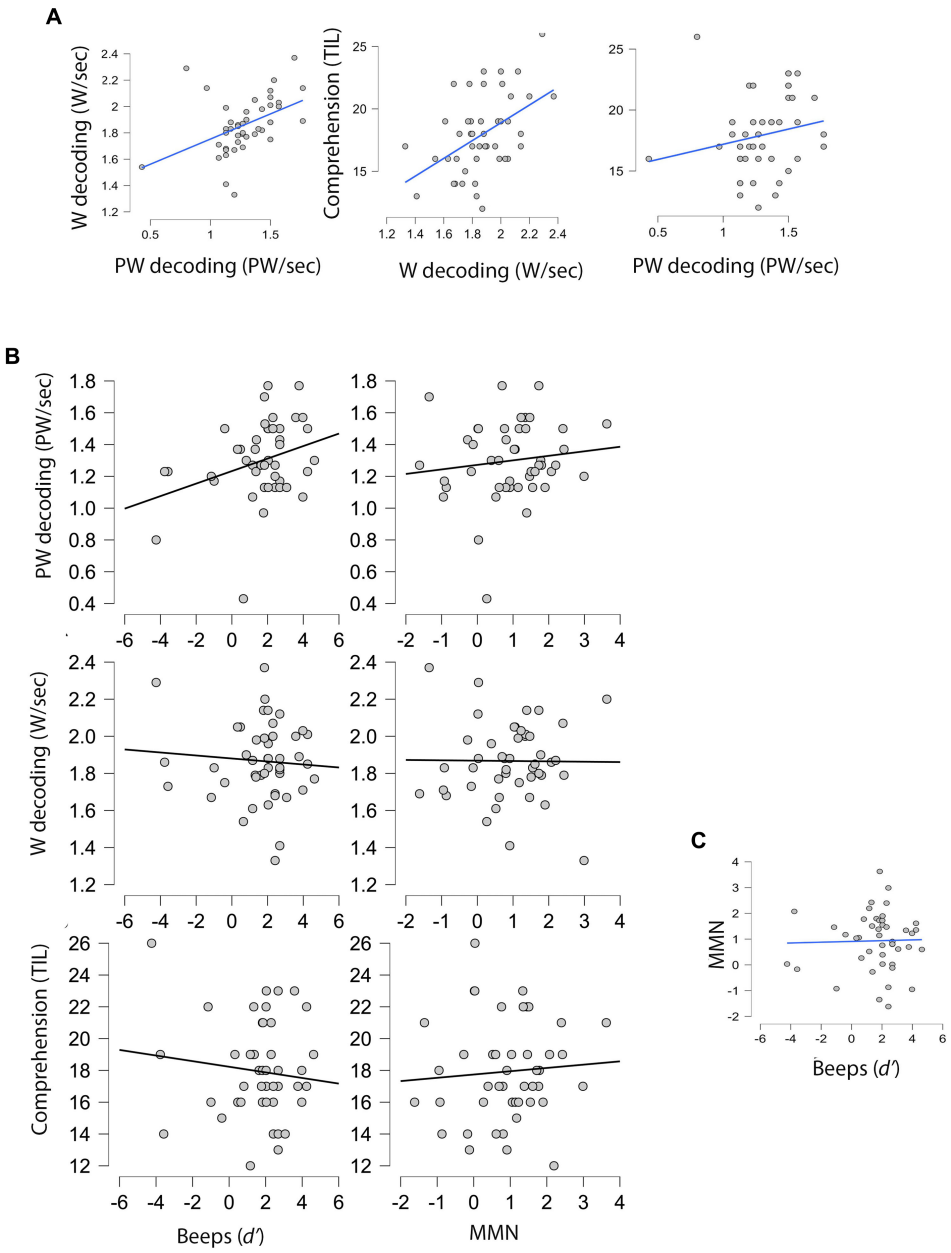
Scatterplots for variable pairs in the main sample (*n* = 46). **(A)** Relations among reading measures (dependent variables). **(B)** Relation of domain-general (Beeps) and speech-specific (MMN) duration perception (predictors) with the three dependent variables. **(C)** Relation between the two predictors. PW, Pseudoword; W, Word.

Figure 3 displays the associations between all variables. As shown in the last plot (section C), the correlation between domain-general and speech-specific duration perception was clearly non-significant, *r*(46) = 0.026, *p* = 0.862, indicating that the two predictors represent different processes.

Table 3 presents the two-predictor multiple regression models (enter method) used to compare domain-general (interval comparison within beep sequences) vs. speech-specific duration perception (MMN paradigm for VOT) on reading measures (decoding of words, pseudowords, and reading comprehension). Only the model for pseudoword reading had a marginal result. Pseudoword reading was significantly predicted by one modality of duration perception, specifically by domain general duration perception. After removing the non-significant predictor from the model, it became significant (see Table 3, one-predictor model).

**Table 3:** Multiple regression models for reading measures with speech-specific (MMN) and domain-general duration perception (Beeps) as predictors (*N* = 46).

Regression model	B	*β*	*t*	*P*	Adjusted *R^2^*	*F*	*p*
Word decoding MMN Beeps	−0.002 −0.008	−0.008 −0.075	−0.053 −0.494	0.958 0.654	−0.041	0.124	0.884
Pseudoword decoding MMN Beeps	0.027 0.039	0.120 0.310	0.837 2.158	0.407 0.037	0.071	2.727	0.077
(One-predictor model) Beeps	0.039	0.313	2.188	0.034	0.078	4.787	0.034
Reading comprehension MMN Beeps	0.215 −0.180	0.076 −0.112	0.503 −0.742	0.671 0.462	−0.028	0.392	0.678

### 3.4. Domain-general duration perception and reading with *n* = 96

**Figure 4:**
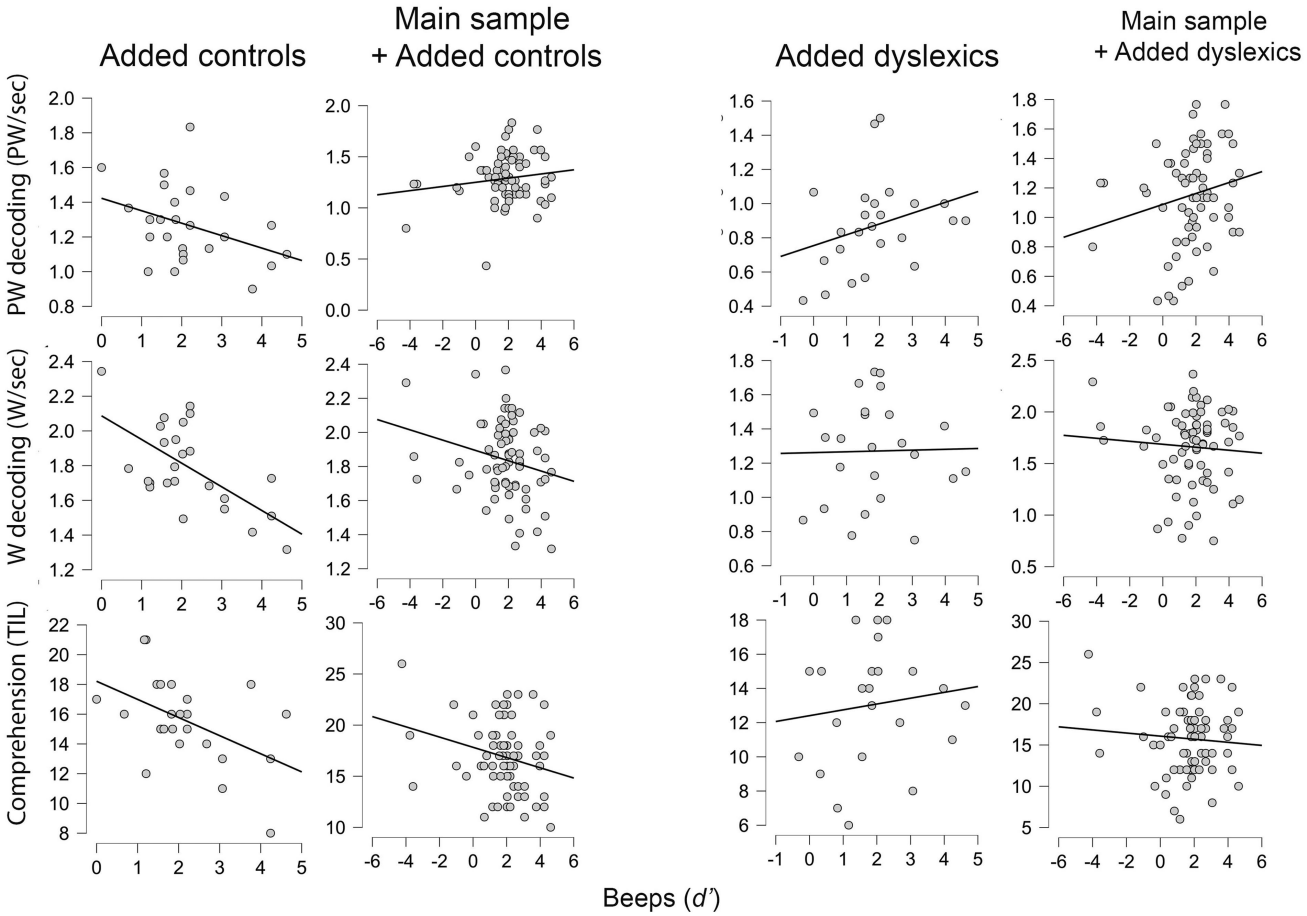
Scatterplots for the relation between domain-general duration perception (Beeps) and the three dependent variables (reading measures) in the enlarged sample (*n* = 96) considering different subgroups. Data from added controls (*n* = 26) and added dyslexics (*n* = 24) pertains to Catronas et al. (2023). Main sample refers to the current study (*n* = 46). PW, Pseudoword; W, Word.

The complementary analysis with an extended sample of *n* = 96 (46 controls from main study, 26 controls and 24 dyslexics from our previous study) continued to show evidence of an association between domain-general duration perception and pseudoword reading. Nevertheless, evidence was restricted to the dyslexic group, as we found after examining interactions (Figure 4). Note that this analysis did not include speech-specific duration perception because extra data was obtained from a previous study of ours (Catronas et al., 2023), in which only this modality of duration perception was assessed. The 26 + 24 participants from Catronas et al. (2023) were already classified as controls vs. dyslexics, based on an extensive evaluation of reading profiles, and so we skipped the inspection of reading measures. Descriptive values for these measures and the beeps task may be found in Supplementary Table SA1 from Supplementary materials. The detailed results of the complementary analysis follow below.

Regarding regression models, only the one for pseudoword decoding showed a significant role of duration perception as predictor. However, all models (Table 4) highlighted significant interactions between duration perception (beeps) and group, between duration perception and study, as well as a significant predictive role of group. The latter reflects the lower scores obtained by dyslexics in reading tasks, as documented in the Supplementary Tables SB, SC, SD.

**Table 4:** Multiple regression models for reading measures with domain-general duration perception (Beeps) as predictor [*N* = 96, 46 from current study, 26 controls and 24 dyslexics collected for Catronas et al., 2023].

Regression model	B	*β*	*t*	*P*	Adjusted *R^2^*	*F*	*p*
Word decoding					0.549	23.69	< 0.001
Beeps	−0.008	−0.037	−0.442	0.660			
Study	0.211		1.788	0.077			
Group*	−0.829		−5.973	< 0.001			
Beeps x study*	−0.130		−2.769	0.007			
Beeps x group*	0.143		2.465	0.016			
Pseudoword decoding							
Beeps*	0.039	0.209	2.153	0.034	0.403	13.17	< 0.001
Study	0.205		1.739	0.086			
Group*	−0.683		−4.929	< 0.001			
Beeps x study*	−0.111		−2.371	0.020			
Beeps x group*	0.135		2.334	0.022			
Reading comprehension							
Beeps	−0.176	−0.072	−0.810	0.420	0.502	18.94	< 0.001
Study	−0.325		−0.230	0.819			
Group*	−6.863		−4.132	<0.001			
Beeps × study*	−1.382		−2.461	0.016			
Beeps × group*	1.749		2.510	0.014			

The asterisk indicates significant predictors.

To clarify the meaning of the interactions, we ran separate correlations for controls and dyslexics from Catronas et al. (2023), and also for the combinations of these groups with the main sample (*n* = 46). For combinations, we ran partial correlations, namely by controlling for study in the main sample + added controls combination and controlling for study and group in the main sample + added dyslexics combination. One-tailed correlational tests (Table 5) showed positive associations between duration perception and pseudoword reading in the dyslexic group alone, and also when both dyslexics (*n *= 24) and the main sample of this study (*n* = 46) were considered. In contrast, neither the added controls (*n* = 26) nor these gathered with the main sample showed significant associations with pseudoword reading. Correlations engaging word decoding and reading comprehension were null in all cases.

**Table 5:** Correlations between reading measures and general-domain duration perception (beeps) in Catronas et al. (2023) data.

	Added controls	Added dyslexics	Main + added controls^b^	Main + added dyslexics^b^
Reading measures	Beeps			
Word decoding	−0.624	0.021	−0.111^a^	−0.020^a^
Pseudoword decoding	−0.371	0.278*^a^	0.024^a^	0.0.153*^a^
Reading comprehension	−0.480	0.111	−0.145^a^	−0.008^a^

a The non-parametric Kendall’s tau test was added due to normality violation.

b Controlled for study.

All one-tailed tests for positive correlations; *p < 0.05.

## 4. General discussion

In the present study we wanted to compare domain-general with speech-specific duration perception as statistical predictors of reading in adults, this including word decoding, pseudoword decoding, and reading comprehension. We predicted that associations would favor pseudoword decoding, whatever the duration perception modality (domain-general vs. speech-specific) with the strongest predictive power. To that end, we ran one regression model with the two time-related predictors for each of the three reading skills, and we carried out a complementary analysis with an enlarged sample adding 26 neurotypical +24 dyslexic adults to further investigate the associations of domain-general duration perception with reading. Based on available studies, which have considered the two modalities separately, we also predicted that domain-general would be more important to reading than speech-specific duration perception.

In line with our predictions, pseudoword decoding was the only reading skill that showed an association with duration perception. This makes sense in light of the idea that, mostly in adults, pseudoword – unlike word reading – requires grapheme-to-phoneme conversion processes (Pammer, 2014). Grapheme-to-phoneme conversion relies heavily on phonological encoding (Baldeweg et al., 1999), which seems key to explain the relation between duration perception and reading, whether it is based on entrainment or duration perception for speech sounds. Unlike pseudowords, words tend to become recognized holistically, as visual word forms, as reading acquisition progresses (Soares et al., 2022), while phonological processing becomes less important. The same would apply to reading comprehension, which is related to words. This developmental change in reading routes may explain why previous studies found associations with word decoding and even reading comprehension (e.g., Corriveau et al., 2007; Plourde et al., 2017; Casini et al., 2018), while we found none.

Pseudoword decoding was associated with domain-general duration perception in the main sample (*n* = 46), in the added sample of dyslexics (*n* = 24), but not in the added sample of controls. We have currently no definite explanation for this, but one possibility relates to the characteristics of Catronas et al. (2023) study, which might have affected controls’ performance more than that of dyslexics. This previous study of ours was remarkably lengthy (two sessions, 2.5 hour each approximately). In the first session, participants underwent an extensive evaluation of their reading profiles (including 3DM and TIL) and, in the second, they performed a series of experimental tasks that included eye-tracking data collection (they had to sit still for a long time). It is likely that all participants experienced fatigue, but, due to their clinical history, dyslexics tend to be more familiar with evaluation sessions and they are also more likely to have developed compensatory strategies to keep focus on the tasks. Therefore, we can consider the possibility that controls, more than dyslexics, suffered with the novelty and strain arising from the circumstances of data collection sessions. As a consequence, their performance may have been more disrupted in different ways, dissolving the positive association between pseudoword decoding and domain-general duration perception. Note that, although the added controls outperformed the added dyslexics in all tasks, they underperformed participants from the main sample in reading comprehension (Supplementary Tables, Supplementary Figure SD).

Regarding our main question – which duration perception modality predicts reading more strongly – we found that domain-general is more relevant to reading than speech-specific duration perception, at least for the tasks we used here. Our findings are in line with the available literature, showing weaker and less consistent results for the latter (speech-specific). They are also in line with the idea that the efficient processing of durations in speech may be less important than, for instance, the processing of spectral and pitch-related properties (Kraus et al., 1996; Baldeweg et al., 1999).

Our findings highlight the need for further research in key areas. First, it will be important to make direct comparisons between children and adults to determine with proper control whether duration perception is linked to pseudowords only in adults but also to words in children, in which grapheme-to-phoneme conversion is expected to be more widespread. This will allow a better understanding on the role of phonological encoding, as we hypothesized here. Second, our results were not totally consistent across studies, in that neurotypical adults in the current study showed a positive association between duration perception and pseudoword reading, while their counterparts in Catronas et al. (2023) did not. We proposed an explanation based on marked differences across study settings, but this will require further testing, namely by gathering a large sample of neurotypical adults for a single study. Our results suggest that factors other than the ability to perceive duration-related properties of vowels and consonants should be given priority. The current state of the art points to entrainment-to-speech abilities as a strong candidate to explain the importance of domain-general duration perception (Casini et al., 2018), even though it is not clear yet why these abilities are relevant for non-strictly periodic auditory input like speech (Vanden Bosch der Nederlanden et al., 2020). The possibility that entrainment to speech sounds may not require a strict beat-based time in the stimuli is in line with developmental approaches suggesting that a quasi-rhythmic temporal skeleton is enough to allow the infant brain to entrain and to form expectancies about when the next auditory event should occur, thus increasing encoding and, later, the prediction of the event itself (Hämäläinen et al., 2012). Future research on the role of both entrainment and the periodicity of entrainment targets is, thus, priority.

Apart from entrainment mechanisms, alternative explanations for the superior relevance of domain-general duration perception are plausible and deserve further investigation. One may be that the base lengths we used in the two modalities are quite different: for domain-general duration perception we were dealing with hundreds of ms, while for speech specific the intervals to discriminate were much shorter. To address this possibility, a common base length could be looked for, e.g., using processed speech with length contrasts similar to those we used in the beeps task or, perhaps in a more ecological approach, create non-speech analogs (two different timbres, mimicking consonant and vowel) of syllables. A second alternative explanation could be that perceptual skills other than length-related ones are involved in VOT contrasts, for instance, the ability to detect amplitude increases during consonant release. Controlling these and/or non-strictly temporal acoustic features in voicing contrasts would certainly be a valuable contribution. Another point to consider is that the interval comparison task we used to measure duration perception may also engage temporal processing skills. While judging whether the sequence speeded up or slowed down, participants not only had to judge each interval, but also decide on which interval came first – the shortest or the longest, and this relates to ordering events (time intervals as sequential events).This indicates that the task we used was not pure (at least in theory) – meaning that it was not solely about perceiving durations and, thus, that the associations we found may be, in part, associations between temporal processing and pseudoword reading. In future studies, it might be useful to investigate this further by employing time estimation instead of speed-up vs. slow-down judgments, or by running additional tasks specifically designed to assess pure temporal processing and then controlling for this skill when analyzing our beeps task. Finally, we should stress the fact that we made complementary analysis to test the link between domain-general duration perception and reading, but we did not do the same for speech-specific duration perception and its null association with reading. Therefore, even though the association was very weak for the latter, we cannot be sure that values would change with a larger sample. Despite these potential issues, we should note that the stimuli we used for the two modalities shared a common ground, namely the fact of eliciting discrimination between shorter and longer auditory events, and not, for instance, one relying on discrimination and the other on estimation.

Our results strengthened the idea that encoding the durations of speech sounds (how long each sound lasts, speech-specific duration perception) might not be as crucial as using domain-general duration perception skills to better encode speech sounds. This has pedagogical and theoretical implications, of which we highlight a few. For example, educational programs could incorporate duration perception tasks into literacy interventions to target and enhance this specific aspect of duration perception in individuals struggling with grapheme-to-phoneme conversion problems, leading to improved reading outcomes. Moreover, training exercises focused on interval estimation, discrimination and comparison tasks might help improve phonological encoding, thus not only enhancing reading proficiency, but also speech perception as well. These exercises could be integrated into existing phonics-based curricula to enhance phonological awareness and decoding skills. Also, schools and educational institutions could incorporate assessments of domain-general duration perception into their diagnostic tools to identify potential areas of difficulty in phonological processing. This information could then be used to customize interventions for students with specific reading challenges. Moreover, our study bridges the gap between time perception, typically approached by cognitive psychologists, and reading skills, with which educators are also concerned. This cross-disciplinary insight could encourage collaboration between cognitive psychologists and educators and lead to the development of innovative teaching methods that leverage insights from cognitive psychology to enhance reading instruction. Longitudinal studies tracking the development of duration perception skills from childhood to adulthood could provide valuable contributions to designing these novel methods, by delimiting duration perception skills in each age range and thus target time perception adequately across the life span. From a broader viewpoint, the outcome of this study is consistent with the possibility that entrainment to speech is key to optimize phonological encoding, in line with the Temporal Sampling Framework.

Despite its limitations, our study contributed to strengthen the hypothesis that time perception is linked to reading via phonological encoding and provides reliable evidence suggesting that optimal encoding of speech sound durations may be less relevant than simply encoding speech sounds.

## Data availability statement

The datasets presented in this study can be found in online repositories. The names of the repository/repositories and accession number(s) can be found at: https://osf.io/eahsm/?view_only=83531bd7de6b48008c3dcd2320876afb.

## Ethics statement

The studies involving humans were approved by Faculty of Psychology and Educational Sciences at University of Porto (Ref. 2022/01-10). The studies were conducted in accordance with the local legislation and institutional requirements. The participants provided their written informed consent to participate in this study.

## Author contributions

AB, DC, JS, VF, NT, and SS: conceptualization and writing. SS: methodology, supervision, administration, and funding acquisition. DC and SS: software. AB, JS, and SS: analysis. AB and DC: investigation. AB: data curation. AB and SS: visualization. All authors contributed to the article and approved the submitted version.

## Funding

This research was funded by the Portuguese Foundation for Science and Technology (FCT), grant numbers CPUP UIDB/00050/2020 and PTDC/PSI-GER/5845/2020.

## Conflict of interest

The authors declare that the research was conducted in the absence of any commercial or financial relationships that could be construed as a potential conflict of interest.

## Publisher’s note

All claims expressed in this article are solely those of the authors and do not necessarily represent those of their affiliated organizations, or those of the publisher, the editors and the reviewers. Any product that may be evaluated in this article, or claim that may be made by its manufacturer, is not guaranteed or endorsed by the publisher.

## Supplementary material

The Supplementary material for this article can be found online at: https://www.frontiersin.org/articles/10.3389/fnhum.2023.1241589/full#supplementary-material

## Footnotes

^1^ https://www.fon.hum.uva.nl/praat/
