# Supplementary material for: Domain-general but not speech-specific auditory duration perception predicts pseudoword reading in adults
Source: Front Hum Neurosci. 2023 Sep 14;17:1241589. doi: 10.3389/fnhum.2023.1241589 (PMC10539623; doi:10.3389/fnhum.2023.1241589)
Supplement: Supplementary file 1 [file Data_Sheet_1.docx]

Supplementary Material

# Supplementary Figures and Tables

## Supplementary Table A1. Descriptives for reading tests and general domain duration perception across studies and groups

|  | | | | | | | | | | | | | | | | | | | | | | | | | |
| --- | --- | --- | --- | --- | --- | --- | --- | --- | --- | --- | --- | --- | --- | --- | --- | --- | --- | --- | --- | --- | --- | --- | --- | --- | --- |
|  | | **3dmPW_speed** | | | | | | **3dmPALspeed** | | | | | | **Beeps** | | | | | | **TIL** | | | | | |
|  | | **s3e4c** | | **st2c** | | **st3e4d** | | **s3e4c** | | **st2c** | | **st3e4d** | | **s3e4c** | | **st2c** | | **st3e4d** | | **s3e4c** | | **st2c** | | **st3e4d** | |
| Valid |  | 26 |  | 46 |  | 24 |  | 26 |  | 46 |  | 24 |  | 26 |  | 46 |  | 24 |  | 26 |  | 46 |  | 23 |  |
| Miss. |  | 0 |  | 0 |  | 0 |  | 0 |  | 0 |  | 0 |  | 0 |  | 0 |  | 0 |  | 0 |  | 0 |  | 1 |  |
| Mean |  | 1.269 |  | 1.299 |  | 0.872 |  | 1.790 |  | 1.866 |  | 1.270 |  | 2.162 |  | 1.671 |  | 1.865 |  | 15.077 |  | 17.935 |  | 11.391 |  |
| SD |  | 0.215 |  | 0.243 |  | 0.265 |  | 0.242 |  | 0.208 |  | 0.297 |  | 1.111 |  | 1.940 |  | 1.280 |  | 3.334 |  | 3.108 |  | 2.291 |  |
| Min |  | 0.900 |  | 0.433 |  | 0.433 |  | 1.317 |  | 1.333 |  | 0.750 |  | 0.000 |  | -4.241 |  | -0.319 |  | 10.000 |  | 12.000 |  | 6.000 |  |
| Max |  | 1.833 |  | 1.767 |  | 1.500 |  | 2.342 |  | 2.367 |  | 1.733 |  | 4.624 |  | 4.624 |  | 4.624 |  | 22.000 |  | 26.000 |  | 15.000 |  |
|  | | | | | | | | | | | | | | | | | | | | | | | | | |

*Note.* 3dmPW_speed = pseudoword decoding; 3dmPALspeed = word decoding; TIL = reading comprehension test; st2c = main study presented here, n = 46; st3e4c = data from control participants in Catronas et al. (2023); st3e4d = data from dyslexic participants in Catronas et al. (2023).

**Supplementary Table B1.** Anova for word decoding per study and group

|  | | | | | | | | | | | | | |
| --- | --- | --- | --- | --- | --- | --- | --- | --- | --- | --- | --- | --- | --- |
| **Cases** | | **Sum of Squares** | | **df** | | **Mean Square** | | **F** | | **p** | | **η²** | |
| groupSt |  | 5.894 |  | 2 |  | 2.947 |  | 49.733 |  | < .001 |  | 0.519 |  |
| Residuals |  | 5.452 |  | 92 |  | 0.059 |  |  |  |  |  |  |  |
|  | | | | | | | | | | | | | |
| *Note.*  Type III Sum of Squares; groupst = variable with three levels: st2c = main study presented here, n = 46; st3e4c = data from control participants in Catronas et al. (2023); st3e4d = data from dyslexic participants in Catronas et al. (2023). | | | | | | | | | | | | | |

**Supplementary Table B3.** Post-hocs for word decoding per study and group

|  | |  | | **Mean Difference** | | **SE** | | **t** | | **p_tukey_** | |
| --- | --- | --- | --- | --- | --- | --- | --- | --- | --- | --- | --- |
| s3e4c |  | st2c |  | -0.076 |  | 0.060 |  | -1.250 |  | 0.427 |  |
|  |  | st3e4d |  | 0.520 |  | 0.070 |  | 7.471 |  | < .001 |  |
| st2c |  | st3e4d |  | 0.595 |  | 0.061 |  | 9.713 |  | < .001 |  |
|  | | | | | | | | | | | |
| *Note.*  P-value adjusted for comparing a family of 3; st2c = main study presented here, n = 46; st3e4c = data from control participants in Catronas et al. (2023); st3e4d = data from dyslexic participants in Catronas et al. (2023). | | | | | | | | | | | |

**Supplementary Table C1.** Anova for pseudoword decoding per study and group

|  | | | | | | | | | | | | | |
| --- | --- | --- | --- | --- | --- | --- | --- | --- | --- | --- | --- | --- | --- |
| **Cases** | | **Sum of Squares** | | **df** | | **Mean Square** | | **F** | | **p** | | **η²** | |
| groupSt |  | 3.107 |  | 2 |  | 1.554 |  | 26.386 |  | < .001 |  | 0.365 |  |
| Residuals |  | 5.417 |  | 92 |  | 0.059 |  |  |  |  |  |  |  |
|  | | | | | | | | | | | | | |
| *Note.*  Type III Sum of Squares; *Note.*  Type III Sum of Squares; groupst = variable with three levels: st2c = main study presented here, n = 46; st3e4c = data from control participants in Catronas et al. (2023); st3e4d = data from dyslexic participants in Catronas et al. (2023). | | | | | | | | | | | | | |

**Supplementary Table C2.** Post-hocs for word decoding per study and group

|  | |  | | **Mean Difference** | | **SE** | | **t** | | **p_tukey_** | |
| --- | --- | --- | --- | --- | --- | --- | --- | --- | --- | --- | --- |
| s3e4c |  | st2c |  | -0.032 |  | 0.060 |  | -0.529 |  | 0.857 |  |
|  |  | st3e4d |  | 0.394 |  | 0.069 |  | 5.688 |  | < .001 |  |
| st2c |  | st3e4d |  | 0.426 |  | 0.061 |  | 6.977 |  | < .001 |  |

*Note.*  P-value adjusted for comparing a family of 3; st2c = main study presented here, n = 46; st3e4c = data from control participants in Catronas et al. (2023); st3e4d = data from dyslexic participants in Catronas et al. (2023).

**Supplementary Table D1.** Anova for reading comprehension per study and group

| \| **Cases** \| \| **Sum of Squares** \| \| **df** \| \| **Mean Square** \| \| **F** \| \| **p** \| \| **η²** \| \| \| --- \| --- \| --- \| --- \| --- \| --- \| --- \| --- \| --- \| --- \| --- \| --- \| --- \| --- \| \| groupSt \|  \| 665.177 \|  \| 2 \|  \| 332.588 \|  \| 36.949 \|  \| < .001 \|  \| 0.445 \|  \| \| Residuals \|  \| 828.129 \|  \| 92 \|  \| 9.001 \|  \|  \|  \|  \|  \|  \|  \| \|  \| \| \| \| \| \| \| \| \| \| \| \| \| \| \|  \| \| \| \| \| \| \| \| \| \| \| \| \| \| |
| --- | --- | --- | --- | --- | --- | --- | --- | --- | --- | --- | --- | --- | --- | --- | --- | --- | --- | --- | --- | --- | --- | --- | --- | --- | --- | --- | --- | --- | --- | --- | --- | --- | --- | --- | --- | --- | --- | --- | --- | --- | --- | --- | --- | --- | --- | --- | --- | --- | --- | --- | --- | --- | --- | --- | --- | --- | --- | --- | --- | --- | --- | --- | --- | --- | --- | --- | --- | --- | --- | --- |

*Note.*  Type III Sum of Squares; groupst = variable with three levels: st2c = main study presented here, n = 46; st3e4c = data from control participants in Catronas et al. (2023); st3e4d = data from dyslexic participants in Catronas et al. (2023).

**Supplementary** Table D2. Post-hocs for reading comprehension per study and group

|  | |  | | | **Mean Difference** | | | | | | **SE** | | **t** | | **p_tukey_** | |
| --- | --- | --- | --- | --- | --- | --- | --- | --- | --- | --- | --- | --- | --- | --- | --- | --- |
| s3e4c |  | st2c | |  | -2.858 | | | | |  | 0.736 |  | -3.882 |  | < .001 |  |
|  |  | st3e4d | |  | 3.686 | | | | |  | 0.859 |  | 4.291 |  | < .001 |  |
| st2c |  | st3e4d | |  | 6.543 | | | | |  | 0.766 |  | 8.540 |  | < .001 |  |
|  | | | | | | | | | | | | | | | | |
| *Note.*  P-value adjusted for comparing a family of 3; st2c = main study presented here, n = 46; st3e4c = data from control participants in Catronas et al. (2023); st3e4d = data from dyslexic participants in Catronas et al. (2023). | | | | | | | | | | | | | | | | |
| **Supplementary** Table E1. Anova for general-domain duration perception per study and group | | | | | | | | | | | | | | | | |
| **Cases** | | | **Sum of Squares** | | | **df** | | **Mean Square** | | **F** | | **p** | | **η²** | |  |
| groupSt |  | | 4.136 | |  | 2 |  | 2.068 |  | 0.800 |  | 0.452 |  | 0.017 |  |  |
| Residuals |  | | 237.804 | |  | 92 |  | 2.585 |  |  |  |  |  |  |  |  |
|  | | | | | | | | | | | | | | | |  |
| *Note.*  Type III Sum of Squares; groupst = variable with three levels: st2c = main study presented here, n = 46; st3e4c = data from control participants in Catronas et al. (2023); st3e4d = data from dyslexic participants in Catronas et al. (2023).  **Supplementary** Table E2. Post-hocs for general-domain duration perception per study and group   \|  \| \|  \| \| **Mean Difference** \| \| **SE** \| \| **t** \| \| \| **p_tukey_** \| \| --- \| --- \| --- \| --- \| --- \| --- \| --- \| --- \| --- \| --- \| --- \| --- \| \| s3e4c \|  \| st2c \|  \| 0.505 \|  \| 0.399 \|  \| 1.264 \| \|  \| 0.419 \|  \| \|  \|  \| st3e4d \|  \| 0.311 \|  \| 0.459 \|  \| 0.677 \| \|  \| 0.778 \|  \| \| st2c \|  \| st3e4d \|  \| -0.194 \|  \| 0.405 \|  \| -0.479 \| \|  \| 0.881 \|  \| \|  \| \| \| \| \| \| \| \| \| \|  \| \| \| \| \| \| \| \| \|   *Note.*  P-value adjusted for comparing a family of 3; st2c = main study presented here, n = 46; st3e4c = data from control participants in Catronas et al. (2023); st3e4d = data from dyslexic participants in Catronas et al. (2023). | | | | | | | | | | | | | | | |  |

## Supplementary Figures


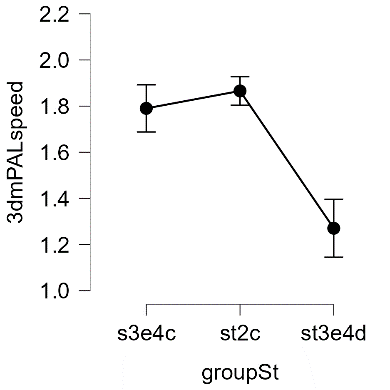


**Supplementary Figure B1.** Pseudoword decoding per study and group


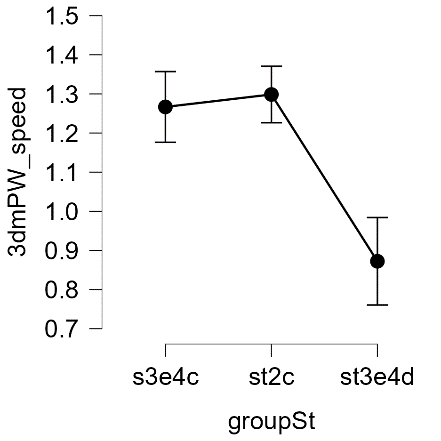


**Supplementary Figure C1.** Word decoding per study and group


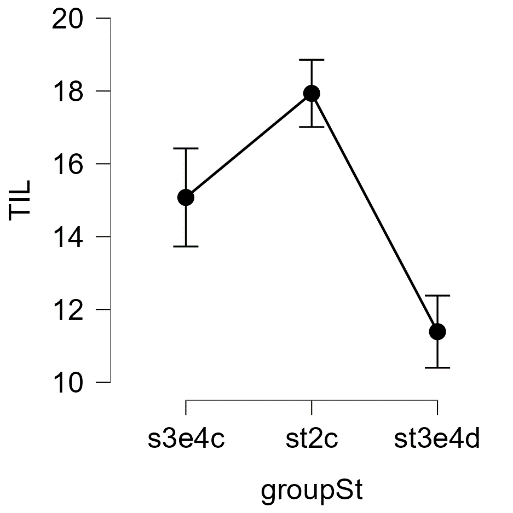


**Supplementary Figure D1.** Reading comprehension per study and group


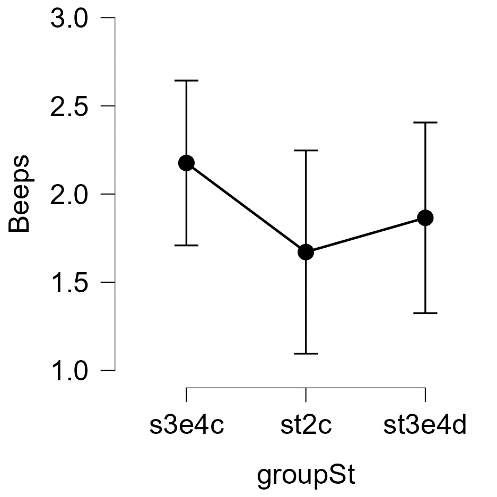


**Supplementary Figure E1.** Duration perception per study and group
